# Cognitive and motor inhibition in balance-related tasks: task-specific associations with executive and physical functions in young and older adults

**DOI:** 10.1038/s41598-026-44189-x

**Published:** 2026-03-17

**Authors:** Eunyoung Kwag, Wiebren Zijlstra

**Affiliations:** Institute of Movement and Sport Gerontology, German Sport University Cologne, Am Sportpark Müngersdorf 6, 50933 Cologne, Germany

**Keywords:** Cognitive control, Balance, Inhibition, Cognitive-motor interaction, Compensatory processes, Human behaviour, Cognitive ageing, Cognitive control, Problem solving

## Abstract

This study investigates age-related differences in relations between inhibitory control in balance-related tasks (BRTs) and executive and physical functions. Correlations between effects of cognitive and motor inhibition in two BRTs and performance on general tests assessing inhibition and other executive functions, as well as associations between performance of the BRTs were explored in 26 young and 46 older adults (YA: 26 ± 4, OA: 70 ± 4 years). Multiple linear regression evaluated BRT-performance using predictors from general tests of executive and physical functions. Significant age-related differences were observed in most general tests. In YA, cognitive inhibition in the BRT correlated with reaction-time and failure-rate in Go/no-go and Stop-signal-tests, while motor inhibition showed a low non-significant correlation with stop-signal reaction-time in Stop-signal-test. These patterns were not observed in OA. In YA and OA, correlations between the BRTs were low and non-significant, and, executive functions, rather than physical functions, partly associated with BRT-performance. The differential associations of cognitive and motor inhibition in the BRTs with general inhibition tests and the lack of correlations between the BRTs supports the notion that the BRTs comprise different aspects of inhibitory control. The absence of similar results in OA may reflect compensatory processes and age-related changes in cognitive functions.

**Supplementary Information:**

The online version contains supplementary material available at 10.1038/s41598-026-44189-x.

## Introduction

Age-related changes in functioning can limit activities of daily living and increase the risk of accidents among older adults (OA). Safe mobility in complex everyday life requires the integration of intact physical and cognitive functions. For example, navigating a crowded area safely and efficiently necessitates inhibiting irrelevant distractions while focusing on relevant information. When encountering a sudden obstacle, individuals must quickly stop ongoing or imminent steps to prevent potential accidents. These conditions, which require simultaneous coordination of cognitive and physical performance, may pose heightened risks for OA, particularly for those with cognitive and physical deficits^1,2^.

The inhibitory deficit hypothesis posits that OA are prone to inefficient inhibitory processes, which affect selective attention and subsequently lead to decreased cognitive performance in various tasks^3^. However, age-related changes in inhibitory control vary depending on task-specific features and the type of inhibition^4–6^, suggesting that inhibitory control does not represent a unitary structure. Evidence also indicates that poor inhibitory control is associated with decreased balance performance and/or increased fall risk in OA^7,8^, which highlights the importance of understanding inhibition-related processes within the context of balance-related tasks (BRTs). However, the relations between inhibitory control and balance performance remain unclear as tasks used to assess inhibition have mostly involved button pressing activities performed while sitting (e.g., Go/no-go test, Stop-signal test, and Flanker test). A scoping review exploring the performance of BRTs that incorporate inhibitory control found that the few available studies indicate that OA exhibit significant inhibition-induced responses, such as increased response times^9^. However, further investigation is needed due to the varied nature of such tasks, which include different types of BRTs, such as gait or step initiation, combined with different types of inhibition (e.g., cognitive and motor inhibition) and different stimuli.

Building on findings of the scoping review^9^, two novel BRTs, simulating complex and unexpected situations requiring inhibitory control, were developed: a stepping task that incorporates cognitive inhibition and a gait initiation-stop task that incorporates motor inhibition^10–12^. Both BRTs require preparation of a step response, however, whereas the stepping task requires the selection and execution of a correct step in response to a visual stimulus, the gait-initiation stop task requires the successful inhibition of step execution in response to a stop signal. Both BRTs were used to examine effects of age and inhibition on the process of preparing an initial response (preparatory phase) and the execution (or inhibition) of a step response (behavioral phase). In both tasks, performance was assessed using a force plate (to analyze changes in center of pressure (CoP) preceding a step) and a marker-based motion capture system (to analyze the execution of a step). Both BRTs demonstrated significant age-related declines^10–12^. However, it remains unclear whether cognitive and motor inhibition integrated into BRTs associate with cognitive and motor inhibition measured by general inhibition tests, and how performance of the stepping and gait initiation-stop tasks is associated within young adults (YA) and OA.

Therefore, this study aims to develop a better understanding of the underlying features of the two novel BRTs, and their age-related differences. First of all, we examine how inhibition effects on these BRTs relate to general tests of inhibition and other executive functions, in order to evaluate whether the BRTs indeed primarily relate to inhibitory control. Secondly, we investigate relations between the two BRTs in order to determine whether these represent the same or different outcomes. Lastly, we determine the extent to which cognitive and physical functions predict overall performance in the BRTs among YA and OA. We hypothesize that in YA as well as in OA the BRTs relate more to inhibitory control than other executive functions, and that the two BRTs represent different aspects of inhibitory control.

## Methods

This study is part of the project “Investigation of the performance of balance tasks requiring inhibitory control in healthy young and healthy old persons”, which has been approved by the ethics committee of the German Sport University (Nr. 095/2021). All methods were performed in accordance with relevant regulations and guidelines.

### Participants

Healthy young (aged 20–35 years) and older (aged 65–75 years) adults participated in the study (YA: *n* = 26, age 26 ± 4; OA: *n* = 46, age 70 ± 4) after providing written informed consent in accordance with the Declaration of Helsinki. The participants completed brief self-report items assessing fall history, fear of falling, and physical activity.

Inclusion criteria comprised an intact ability to hear and see (with or without assistive devices) and an absence of health conditions that affect mobility and/or balance. Exclusion criteria comprised acute injuries, chronic diseases, sensory impairments, gait and/or balance deficiencies, and the inability to walk without assistive devices. Exclusion criteria were assessed through a questionnaire, which also included other background questions. Additionally, the Montreal-Cognitive-Assessment test was conducted for a cognitive screening; a cut-off score of < 23 was used^13^ as exclusion criterion.

### Measurement process and experimental setup

Measurements were scheduled over two days to avoid cognitive and/or physical fatigue effects. All general tests evaluating executive and physical functions were administered by a single rater in the laboratory on the first day of measurement, whereas the two novel BRTs were measured on a second day, within one week after the first day.

The stepping and gait-initiation stop tasks that incorporate cognitive and motor inhibition, respectively, were conducted in randomized order. The initial posture for each task was to stand on a force plate while focusing on a stimulus presented at the centre of a TV screen (93 × 52 cm). Performance was measured using the force plate (Bertec Corporation, US) at a sampling rate of 1000 Hz and a marker-based 3D-Motion analysis system (Qualysis Motion Capture System, Goteborg, SE). Reflective markers were attached to anatomical points in order to measure positions of body segments with 8 infrared cameras (100 Hz). Visual stimuli of the tasks were developed using custom scripts of MATLAB based on Psychtoolbox-3 (MATLAB, R2022a, MathWorks, Natick, MA, USA).

### Assessment of general tests and balance-related tasks integrating inhibition

#### General tests

Tests assessing executive functions were followed by tests assessing physical functions. Three core components of executive functions were measured^14^: (1) inhibitory control evaluated through web-based tests, including Go/no-go test and Stop-signal test. Performance of the Go/no-go test indicates cognitive inhibition, requiring attentional/interference control^15–17^, while performance of the Stop-signal test indicates motor inhibition, requiring an action cancellation/suppression^18^; (2) cognitive flexibility (paper-and-pencil form): Trail making test^19^ and (3) working memory (tapping cubes): Corsi block test^20,21^. Tests assessing physical functions included: (1) balance test: Berg balance scale^22^, (2) mobility test: modified Timed-up & go (based on^23^) and (3) balance confidence test: Activity-specific balance confidence scale^24^. Detailed information regarding test procedures and variables is provided in Appendix 1. The web-based executive functions tests were developed using custom scripts on the PsyToolkit platform.

#### Balance-related tasks that incorporate inhibition

**Fig. 1:**
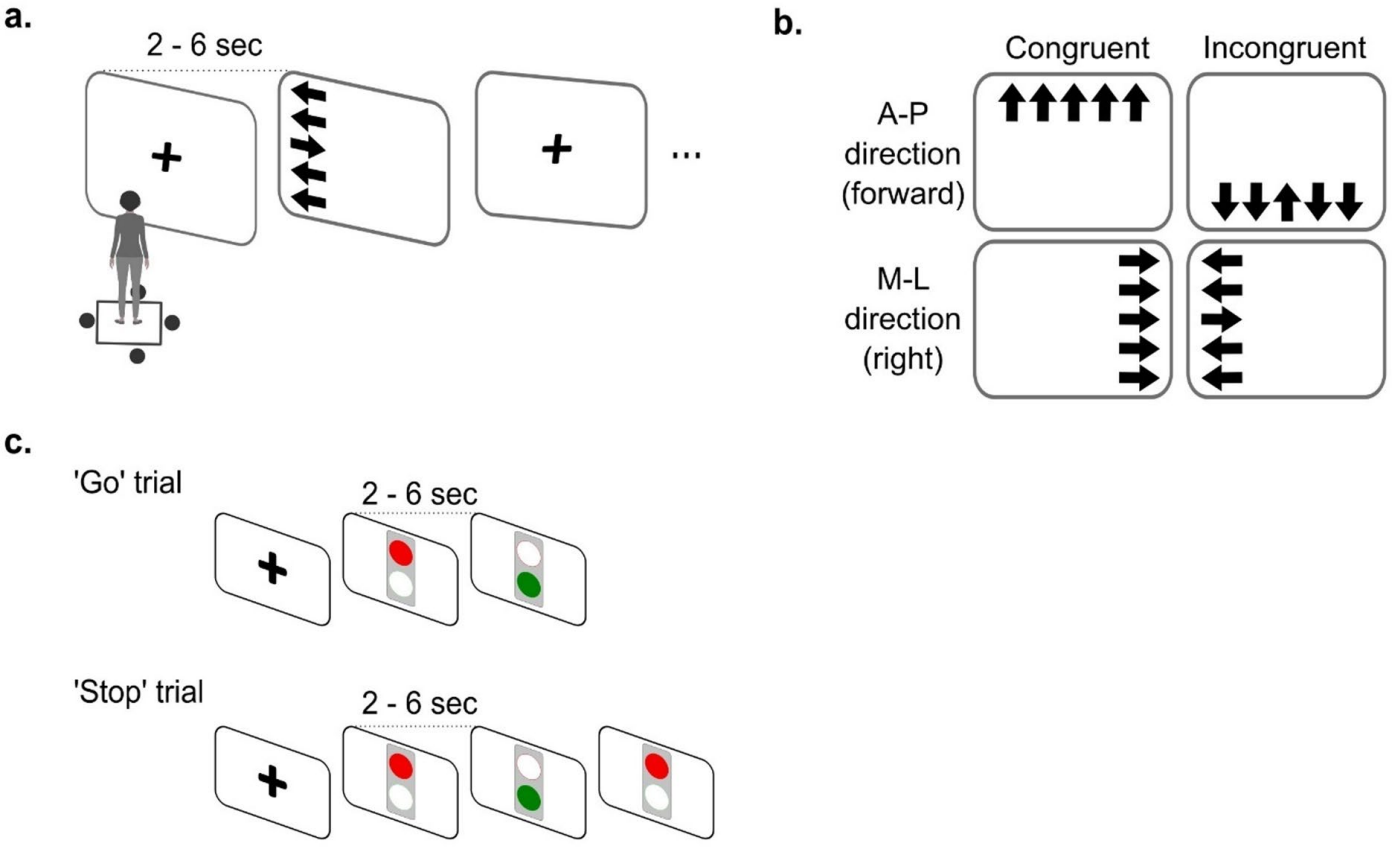
Balance-related tasks that incorporate inhibitory control. (**a**) illustrates the process of the Simon-Flanker stepping task that incorporates cognitive inhibition. After a participant makes a response according to the instruction, and once a stable quiet standing position is assumed on a force plate, a researcher initiates the next stimulus presentation, which was presented within a 52 × 51 cm region centered on the screen. This begins with a display of the (+) symbol, followed by the subsequent arrow stimulus. (**b**) presents an example of stimuli indicating a forward step in the anterior-posterior direction (A-P) and a right step in the medial-lateral direction (M-L) as the correct response, including both congruent and incongruent trials. (**c**) shows the ‘Go’ and ‘Stop’ trials from the gait initiation-stop task that incorporates motor inhibition.

The stepping task that incorporates cognitive inhibition is designed based on combined principles from a Simon- and Flanker-task (i.e., a Simon-Flanker task by Kwag et al.^10^). The task consisted of 20 congruent and 20 incongruent stimulus-response trials, with five of each across four step directions (forward, backward, left, and right). Prior to the task, participants were instructed on how to respond to the stimuli, and all eight cues (four step directions x congruent and incongruent trials) were demonstrated. A participant was required to respond quickly to an ‘arrows’ stimulus which followed a plus signal, by stepping onto an individually normalized target while shifting their weight, then returning to the starting position before the next trial triggered by the researcher (see Fig. 1a for details). A correct response for the Simon-Flanker task was to step into the direction indicated by the middle arrow while ignoring both the location and the direction of the surrounding arrows (see Fig. 1b for example of correct response of forward and right).

The gait initiation-stop task that incorporates motor inhibition consisted of three blocks, with each block comprising 9 ‘Go’ and 3 ‘Stop’ trials in a randomized order. The participant was required to initiate gait promptly when the light turned to green (i.e., ‘Go’ trial) and completely block gait initiation, maintaining an upright standing position on the force plate, when the green signal changed from green to red (i.e., ‘Stop’ trial) in some trials (see Fig. 1c) (for detailed information, see Kwag, Komnik, et al., 2024 ^11^).

### Data analysis

#### General tests

Before data analysis, the failure rate of the stop trials in the Stop-signal test was used as an exclusion criterion to ensure reliability of stop signal reaction time estimates. Participants with a probability of responding on stop trials below 0.25 or above 0.75 were excluded^25^.

#### Balance-related tasks that incorporate inhibition

Participants with missing data in any type of trial (congruent or incongruent) or step direction in the stepping task, due to instability before stimulus presentation or incorrect step responses, were excluded from further analysis. In the gait initiation-stop task, participants with missing data in ‘Stop’ trials, caused by excessively fast or delayed responses (CoP onset < = 125 ms or > = 550 ms), were also excluded.

Data analysis of the BRTs encompassed changes in ground reaction forces and the CoP position from the force plate, as well as step movement from the motion capture system. The ground reaction forces and the CoP signal were filtered using a second-order, recursive Butterworth filter with a cut-off frequency of 5 Hz.

Overall outcome of the stepping task was assessed by the duration in total step execution, calculated as the mean time between presentation of stimulus and touchdown across all congruent and incongruent trials. Effects of cognitive inhibition were analyzed based on changes in CoP onset and CoP duration, which occur prior to the behavioral phase, such as step execution (for detailed information see Data analysis by Kwag, Bachmann, et al., 2024^10^). Effects of cognitive inhibition were calculated as the additional duration due to inhibition, expressed as the ratio of the difference between incongruent and congruent trial times to the congruent trial time.

For the gait initiation-stop task, only trials of comparable difficulty were included to examine overall outcome, success rate, and motor inhibition. To this purpose, the CoP threshold suggested by an ROC-analysis was used^26^, and trials with a CoP shift larger than 34 mm when the stop signal was presented, were excluded from further analysis (for detailed information see Data analyses and Results by Kwag, Komnik, et al.^11^). Success was defined as no forward movement in either foot following the stop signal. Motor inhibition was analyzed by estimating integrals based on the amplitude and timing of maximum posterior CoP displacement. The relative motor inhibition during a ‘Stop’ trial was quantified as: StopGo integrals = 100 x (CoP integrals of a ‘Stop’ trial – mean CoP integrals of all ‘Go’ trials) / (mean CoP integrals of all ‘Go’ trials).

### Statistical analysis

Using G*Power (version 3.1.9.7^27^), an a priori power analysis and sample size estimation were performed based on step reaction times presented by Magnard et al. (2020)^28^. To detect group differences, a total sample size of 32 was calculated, based on an effect size of 1.34, with an alpha level of 0.05 and 95% power. For detecting within-group condition effects, the analysis suggested sample sizes of *n* = 24 for YA and *n* = 42 for OA, based on effect sizes of 0.58 and 0.45, respectively, with an alpha level of 0.05 and 80% power. Given these outcomes, the higher sample size suggestions were chosen to ensure robust detection of within-group condition effects. Assuming a 10% drop-out rate, a minimum of 26 YA and 46 OA were recruited.

Data processing and statistical analysis were carried out using MATLAB software. The variables included for tests assessing executive and physical functions were: (1) Reaction time & (2) Failure rate during No-go signal for Go/no-go test, (3) Stop signal reaction time & (4) Failure rate for Stop-signal test, response time for (5) Trail making test Part A and (6) Part B & (7) the difference (B – A), (8) Forward and (9) Backward product for Corsi block test, the Timed-up & go test at (10) fast & (11) normal walking speeds, (12) the Berg balance scale, and (13) the Activity-specific balance confidence scale. After excluding outliers with a z-score greater than three from each group, the Welch’s t-test is used to compare the means of two groups, and a two-tailed *p*-value is reported.

Spearman correlations were conducted to examine the relations between the general tests and the BRTs, as well as among the BRTs. Correlations examining relations between general inhibition tests and the BRTs, as well as among the BRTs themselves, were controlled for multiple comparisons using the Benjamini-Hochberg false discovery rate procedure. All other exploratory correlations were conducted without correction.

Subsequently, a multiple linear regression was performed using only predictors with correlation coefficients exceeding 0.3^29^, employing a stepwise approach with forward selection and backward elimination at a *p*-value threshold of 0.1 to iteratively refine the model. This approach ensures compliance with model assumptions, such as linearity, and helps prevent overfitting. Regression coefficients are reported with 95% confidence intervals. In addition, bootstrap resampling using 2000 iterations was used to assess model stability given the small sample size by examining variability in predictor selection and adjusted *R*^*2*^. Regression diagnostics, including residual analysis and variance inflation factors, were used to assess model assumptions such as normality, homoscedasticity, and multicollinearity.

Magnitude of correlation coefficients was categorized as follows: very high (0.90 to 1.00), high (0.70 to 0.90), moderate (0.50 to 0.70), low (0.30 to 0.50) and negligible (0.00 to 0.30)^30^. In accordance with recommendations for studies with limited sample sizes^29^, only correlation coefficients exceeding 0.30 were considered ‘meaningful’ for further regression analyses. In the context of regression analysis, the coefficient of determination (R-squared), which ranges from 0 to 1, is considered meaningful when it exceeds 0.15^31,32^.

## Results

After data processing, missing data resulted in the exclusion of eight YA (stepping task: *n* = 3; Stop-signal test: *n* = 5) and six OA (Montreal-Cognitive-Assessment test: *n* = 1; stepping task: *n* = 3; Stop-signal test: *n* = 2; gait initiation-stop task: *n* = 1). Finally, full data sets of 18 YA and 40 OA could be included in the analysis. General background data and physical activity-related characteristics are presented in Table 1.

**Table 1:** Characteristics of the participants.

Characteristic	YA (*n* = 18)		OA (*n* = 40)	
	Mean	SD	Mean	SD
Age (years)	25	4	70	3
Gender (number of females (%))	11 (61)		18 (45)	
Height (cm)	172.6	8.7	172.2	8.3
Mass (kg)	65.0	9.9	76.3	13.1
MoCA	28.3	1.0	27.2	1.5
Individuals who experienced a fall in the past 12 months (yes)	1		8	
Fear of falling (yes)	1		7	
Exercise (hours/week)	6.1	5.6	5.3	6.4
Home-based physical activity (hours/week)	6.2	3.7	9.5	6.2

*MoCA* the montreal cognitive assessment test, *OA* older adults, *SD* standard deviation, *YA* young adults.

### General tests of executive and physical functions

OA required significantly more time to respond to signals and complete executive function tests than YA, while failure rate in the inhibition tests did not differ significantly between YA and OA, with a smaller standard deviation in OA (see Table 2a). The product score of the working memory test was greater in YA, with a higher standard deviation in YA.

**Table 2:** General tests assessing executive and physical functions.

	Test	Variable	YA (*n* = 18)		OA (*n* = 40)		T-test	t	*p*-value
			Mean	SD	Mean	SD	*df*		
a	GNG	Reaction time (ms)	355.6	22.8	452.1	43.5	54.6	11.1	*< 0.001*
		Failure (%)	5.3	6.2	6.0	4.7	26.3	0.5	0.647
	SST	SSRT (ms)	240.0	39.3	274.8	47.5	39.3	2.9	*0.006*
		Failure (%)	38.0	9.2	33.7	5.6	22.8	-1.8	0.080
	TMT	A (s)	16.2	3.7	27.4	5.9	49.9	8.7	*< 0.001*
		B (s)	46.7	10.8	74.8	23.0	55.8	6.3	*< 0.001*
		Difference (s)	30.5	10.2	47.4	20.3	55.1	4.2	*< 0.001*
	CBT	Forward product	53.0	20.8	38.9	9.9	20.5	-2.8	*0.012*
		Backward product	63.8	20.8	43.8	11.9	22.2	-3.8	*0.001*
b	BBT	Total score	56.0	0.0	54.6	1.5	39	-6.0	*< 0.001*
	TUG	Normal (s)	25.5	2.6	27.1	4.0	48.1	1.8	0.078
		Fast (s)	18.3	1.4	20.8	2.2	49.2	5.4	*< 0.001*
	ABC	Total score (%)	96.6	3.1	93.7	5.8	54.1	-2.4	*0.018*
c	The stepping task	CI CoP onset	0.07	0.05	0.05	0.08	45.7	-1.0	0.310
		CI CoP duration	0.20	0.15	0.23	0.13	28.5	0.8	0.436
		Total step execution (ms)	1139.91	96.30	1486.63	199.23	55.6	8.9	*< 0.001*
d	The gait initiation-stop task	MI StopGo integrals	-47.46	18.71	-34.86	19.46	24.9	2.1	*0.046*
		Success (%)	63	0.38	26	0.36	22.6	-3.0	*0.006*

a: tests assessing executive functions; b: tests assessing physical functions; c and d represent novel balance-related tasks integrating cognitive (c) and motor inhibition (d).

*ABC* the activity-specific balance confidence scale, *BBT* the Berg balance scale, *CBT* the Corsi block test, *CI* cognitive inhibition, *CoP* Center of pressure, *GNG* the Go/no-go test, *MI* motor inhibition, *OA* older adults, *SD* standard deviation, *SSRT* stop signal reaction time, *SST* the Stop-signal test, *TMT* the trail making test, *TUG* the Timed-up & go, *YA* young adults.

All physical functioning tests exhibited significantly better performance in YA, except for the Timed-up & go test at normal speed (see Table 2b).

### Balance-related tasks that incorporate inhibition

Total step execution time of the stepping task incorporating cognitive inhibition was significantly longer in OA than in YA. In contrast, cognitive inhibition effects on CoP onset and CoP duration did not differ between age groups (see Table 2c).

In the gait initiation-stop task incorporating motor inhibition, applying a cut-off value from the ROC-analysis led to additional missing data: YA (*n* = 4) and OA (*n* = 5). YA demonstrated both a significantly higher success rate and greater motor inhibition than OA (see Table 2d).

### Inhibitory control during the balance-related tasks

#### Relations between inhibition in balance-related tasks and general inhibition tests

Table 3 presents the correlation analysis between variables that represent cognitive and motor inhibition in the stepping and gait initiation-stop tasks, and general tests assessing inhibition, such as the Go/no-go test and the Stop-signal test. Overall, YA exhibited ‘meaningful’ correlation coefficients (above 0.3) for both cognitive inhibition (CoP onset and CoP duration) and motor inhibition (StopGo integrals), whereas OA did not show comparably high correlation coefficients for either type of inhibition. In YA, cognitive inhibition of the stepping task correlated significantly with reaction time in the Go/no-go test (CoP onset: *r*(16) = 0.719, corrected *p* = .004; CoP duration: *r*(16) = − 0.449, corrected *p* = .084) and failure rate in both Go/no-go test (CoP onset: *r*(16) = − 0.246, corrected *p* = .433; CoP duration: *r*(16) = 0.639, corrected *p* = .017), while motor inhibition of the gait initiation-stop task showed a meaningful correlation with stop signal reaction time in the Stop-signal test (StopGo integrals: *r*(12) = 0.400, corrected *p* = .624), despite the lack of statistical significance.

**Table 3:** Correlation coefficients assessing relations between general inhibition tests and cognitive and motor inhibition in balance-related tasks.

		The stepping task				The gait initiation-stop task	
		CI CoP onset		CI CoP duration		MI StopGo integrals	
		YA	OA	YA	OA	YA	OA
GNG	Reaction time (ms)	***0.719***	-0.278	**-0.449**	0.007	0.042	0.186
	Failure (%)	-0.246	-0.267	***0.639***	-0.192	-0.207	0.090
SST	SSRT (ms)	-0.162	-0.127	-0.117	-0.126	**0.400**	0.170
	Failure (%)	**-0.409**	0.142	**0.478**	-0.116	0.185	0.139

Correlation coefficients greater than 0.3 are indicated in bold, while *p*-values below 0.05 are shown in italics.

*CI* cognitive inhibition, *CoP* Center of pressure, *GNG* the Go/no-go test, *MI* motor inhibition, *OA* older adults, *SSRT* stop signal reaction time, *SST* the Stop-signal test, *YA* young adults.

#### Relations between inhibition in balance-related tasks and other general executive function tests

Appendix 2 shows an explorative correlation matrix between cognitive and motor inhibition variables of the BRTs and other executive functions tests. Generally, no ‘meaningful’ correlation coefficients (exceeding 0.3) were found in YA and OA, except for the variable of cognitive inhibition for CoP duration. In addition to the significant correlations between the cognitive inhibition for CoP duration and general inhibition tests (see Table 3), YA displayed ‘meaningful’ correlation coefficients with cognitive inhibition for CoP duration and both the Forward (*r*(16) = − 0.385, *p* = .115) and Backward (*r*(16) = − 0.334, *p* = .176) Corsi block tests, though not achieving statistical significance. In OA, however, the cognitive inhibition for CoP duration did not correlate with the inhibition tests, but correlated with the Trail making test A (*r*(38) = − 0.329, *p* = .039) and the Forward Corsi block test (*r*(38) = 0.308, *p* = .053).

#### Relations between the two balance-related tasks

Overall performance in the stepping and gait initiation-stop tasks (i.e., total step execution and success rate) did not relate to each other (see Table 4). The motor inhibition (StopGo integrals) exhibited comparatively higher correlation coefficients with the cognitive inhibition for CoP duration in YA (*r*(12) = − 0.407, corrected *p* = .301) and with the total step execution in OA (*r*(33) = 0.330, corrected *p* = .115), though non-significant.

**Table 4:** Correlation coefficients evaluating relations between the balance-related tasks for young and older adults.

		Gait initiation-stop task			
		MI StopGo integrals		Success (%)	
		YA	OA	YA	OA
Stepping task	CI CoP onset	-0.143	-0.281	0.244	-0.020
	CI CoP duration	**-0.407**	0.036	0.060	-0.057
	Total step execution (ms)	0.262	**0.330**	0.032	-0.064

Correlation coefficients greater than 0.3 are indicated in bold, while *p*-values below 0.05 are shown in italics.

*CI* cognitive inhibition, *CoP* Center of pressure, *MI* motor inhibition, *OA* older adults, *YA* young adults.

#### Notable patterns linking physical functions tests and balance-related tasks

In OA, physical functions assessed by general tests, including the Berg balance scale and the Timed-up & go at normal and fast speeds, exhibited more ‘meaningful’ correlation coefficients with BRTs compared to YA (see Appendix 2).

In contrast to YA, who showed a meaningful correlation only between cognitive inhibition for CoP onset and the Timed-up & go at normal speed (*r*(16) = 0.313, *p* = .206) as well as the ABC test (*r*(16) = 0.358, *p* = .145), both cognitive inhibition and motor inhibition in OA were correlated with physical functions tests: CoP onset correlated with the Timed-up & go at fast speed (*r*(38) = − 0.393, *p* = .013) and the Berg balance scale (*r*(38) = 0.393, *p* = .013); CoP duration correlated with the Timed-up & go at normal speed (*r*(38) = − 0.403, *p* = .010); StopGo integrals correlated with the Timed-up & go at normal (*r*(33) = 0.334, *p* = .050) and fast speed (*r*(33) = 0.355, *p* = .037) and the Berg balance scale (*r*(32) = − 0.505, *p* = .002).

Total step execution correlated significantly with the Timed-up & go at fast speed (*r*(16) = 0.494, *p* = .037) and showed a non-significant correlation at normal speed (*r*(16) = 0.346, *p* = .160) in YA, and correlated significantly with the Berg balance scale (*r*(36) = − 0.323, *p* = .048) in OA. In contrast to aforementioned associations, success rate neither showed correlations with physical functions tests in YA nor OA.

### Key determinants influencing overall performance in the balance-related tasks

Table 5 presents the regression analysis using predictors derived from general tests assessing executive and physical functions, limited to variables with ‘meaningful’ correlation coefficients (refer to Appendix 2 for the results of the correlation analysis). The Berg balance scale was excluded for YA, as all participants achieved the maximum score, resulting in zero variability.

**Table 5:** Regression analysis for overall outcome of the balance-related tasks for young and older adults.

			β	Std. error	*t*	*p*-value	95% CI
Total step execution	YA	(Intercept)	356.04	243.45	1.46	0.164	[-162.85, 874.93]
		RT in GNG	2.79	0.67	4.15	*0.001*	[1.36, 4.23]
		TMT-A	-12.88	4.14	-3.11	*0.007*	[-21.71, -4.04]
			*R* ^*2*^	Adjusted *R*^*2*^	*F*-statistic		
			0.62	0.57	*F*(2.15) = 12.4	*0.001*	
	OA	(Intercept)	260.1	306.98	0.85	0.403	[-363.06, 883.34]
		RT in GNG	1.99	0.58	3.44	*0.002*	[0.82, 3.15]
		SSRT	1.14	0.55	2.06	*0.047*	[0.02, 2.26]
			*R* ^*2*^	Adjusted *R*^*2*^	*F*-statistic		
			0.31	0.27	*F*(2.35) = 7.71	*0.002*	
Success	YA	(Intercept)	-1.78	1.36	-1.31	0.217	[-4.76, 1.21]
		RT in GNG	0.01	0.00	2.27	*0.044*	[0.00, 0.02]
		TMT difference	-0.02	0.01	-2.90	*0.014*	[-0.03, -0.01]
			*R* ^*2*^	Adjusted *R*^*2*^	*F*-statistic		
			0.56	0.48	*F*(2.11) = 6.97	*0.011*	

*Β* regression coefficient, *CI* confidence interval, *GNG* the Go/no-go test, *OA* older adults, *R*^*2*^ Coefficient of determination (R-squared), *RT* reaction time, *SSRT* stop signal reaction time, *Std* Standard, *t* t-statistic, *TMT* the trail making test, *YA* young adults.

Overall, better models were observed for YA compared to OA in both BRTs, with particularly strong performance in the stepping task (see Table 5).

Reaction time in the Go/no-go test showed a statistically significant association with performance of the stepping task (total step execution), in both YA (β = 2.79, 95% CI [1.36, 4.23]) and OA (β = 1.99, 95% CI [0.82, 3.15]) and appeared in 87% of bootstrap resamples. Additionally, the Trail making test A significantly predicted total step execution in YA (β = −12.88, 95% CI [− 21.71, − 4.04]), while stop signal reaction time was a significant predictor in OA (β = 1.14, 95% CI [0.02, 2.26]). In bootstrap analyses, the Trail making test A showed a selection stability of 82% in YA, whereas stop signal reaction time showed a selection stability of 65% in OA. The models accounted for 62% of the variance in YA (adjusted *R*^*2*^ = 0.57), whereas in OA it accounted for 31% (adjusted *R*^*2*^ = 0.27). Bootstrap resampling yielded a distribution of adjusted *R*^*2*^ values with medians of 0.62 in YA (95% CI [0.26, 0.84] and 0.29 in OA (95% CI [0.04, 0.55]).

In YA, performance in the gait initiation-stop task showed that reaction time in the Go/no-go test (β = 0.01, 95% CI [0.00, 0.02]) and the Trail making test B-A (β = -0.02, 95% CI [-0.03, -0.01]) were significant predictors, with a coefficient of determination value of 0.56, similar to the stepping task. Reaction time in the Go/no-go test showed a selection stability of 81%, and the Trail making test B-A showed a selection stability of 69%. In OA, no variables showed ‘meaningful’ correlation coefficients, and therefore, regression analysis was not conducted.

Variance inflation factors for all retained predictors were below 2, indicating no problematic multicollinearity, and residual diagnostics showed no substantial deviations from normality or homoscedasticity.

## Discussion

Our study aimed to examine whether inhibitory effects observed in two novel BRTs associate with inhibition measured by general inhibition tests, and whether it is associated with other measures of executive functions (cognitive flexibility and working memory). In addition, the relations between the BRTs incorporating cognitive and motor inhibition were examined. Finally, significant predictors of the performance in the BRTs were investigated among executive and physical functions assessed through general tests in YA and OA. Our results suggest that performance of the BRTs incorporating cognitive and motor inhibition, respectively, indeed specifically requires each type of inhibitory control, rather than other executive functions. In addition, executive functions significantly affect performance of the BRTs, rather than physical functions. A key finding of our study is that, unlike in YA, the OA did not show consistent associations between general inhibition tests and the BRTs, nor could their performance on the BRTs be predicted.

The next sections will first address the specific age-related differences in executive and physical functions assessed by general tests, then discuss the findings in YA and highlight and interpret the different findings in OA. The discussion section ends by addressing strengths and weaknesses of this study, followed by conclusion and an outlook on further studies.

### Age-related differences in general tests assessing executive and physical functions

Participants in the older group of this study seemed to be in optimal physical health, with an average of 5 h and 10 h per week dedicated to exercise and home-based physical activity, and a fall incidence of 20%. This fall incidence is lower than the global average of 27–35% among OA^33,34^. Activity levels of our older participants adhere to the maximal recommended dose of physical activity for OA (150–300 min of moderate-intensity activity weekly), which contrast with the fact that less than 15% ofOA meet these guidelines^35^.

Despite of the optimal physical health in OA, all general tests examining cognitive and physical functions have identified statistically significant age-related differences, except for the failure rate in the Go/no-go and Stop-signal tests, as well as Timed-up and go test at normal speed. Furthermore, OA exhibited overall greater variability, as measured by standard deviation, except in the failure rates and Corsi block test. Reaction time and failure rate in the Go/no-go test in OA align with a previous study using an equal proportion of No-go trials, showing a reaction time of approximately 500 ms and a 6% failure rate^36^. In contrast, YA in the present study responded about 100 ms faster but exhibited a 3%higher failure rate compared to the previous findings^36^, indicating a speed-accuracy tradeoff^37,38^. Stop signal reaction time in YA closely mirror that reported in a previous paper examining the Stop-signal test across the life span (248 ms by Bedard et al.^39^ vs. 240 ms in the current study). However, OA in our study demonstrated better stop signal reaction time (329 ms^39^ vs. 275 ms in the current study). The stringent data processing, which is applied (i.e., excluding data with the failure rate < 25% or > 75%) to ensure a reliable stop signal reaction time^25^, may have contributed to the absence of significant age-related differences in failure rate. Physical functions, as assessed by the Timed-up and go at normal pace, did not show differences between OA and YA. However, YA performed significantly faster at a fast pace.

### The two balance-related tasks capture different aspects of inhibitory control

Theoretical distinctions in inhibitory functions, such as controlling attention versus suppressing inappropriate responses, have led to the use of different terms, including cognitive and motor inhibition^40,41^. Different aspects of inhibitory control have been confirmed through neuroimaging techniques, which reveal that cognitive and motor inhibition, despite superficial similarities, rely on distinct subcortical mechanisms with differences in both location and activation patterns^41–45^. In the current study, we identified that in YA, cognitive inhibition integrated into a BRT is moderately to highly significantly associated with cognitive inhibition (attentional/interference control) measured by the Go/no-go test. In contrast, motor inhibition during a BRT shows a moderate (though non-significant) correlation with motor inhibition (action cancellation/suppression) measured by the Stop-signal test. These findings suggest a specificity of cognitive and motor inhibition as well as a convergence between these specific inhibitory processes integrated into the BRTs and those measured by general tests.

The ‘Unity/Diversity framework’ proposed by Miyake et al., (2000)^46^, a widely regarded model for understanding executive functions, emphasizes the significant intercorrelations among the core components of executive functions while affirming their distinctiveness. In our data of YA, both cognitive and motor inhibition integrated in the BRTs were associated with general inhibition tests. However, they mostly did not show associations with other executive functions, thus indicating a clear divergence within the broader executive function framework. The only exception to this finding was the effect of cognitive inhibition on CoP duration (defined as the interval between CoP onset and heel off). During this phase, postural correction and/or adjustments may be needed^47,48^. Thus, during the CoP duration phase, the interaction between information processing and fine-tuning of postural coordination requires broader executive functions. This suggests the complex interplay of cognitive and physical functions during the CoP duration requires the involvement of complex executive functions.

The interrelated features of executive functions (explained by the ‘Unity/Diversity framework’^46^) may contribute to the well-known ‘task impurity problem’, as general inhibition tests often incorporate lower-level processes, such as attention and processing time, in addition to inhibitory control^49,50^. Using adjusted measures, such as combining accuracy and speed, offers a more effective approach to mitigating task impurity and provides a clearer understanding of specific types of inhibition compared to relying solely on raw variables, such as reaction time^49^. In a corresponding way, the examination of cognitive and motor inhibition within the BRTs in the present study may enhance specificity by accounting for inhibition effects (i.e., incongruent compared to congruent trials, and stop compared to go trial). This is supported by the finding that no significant associations were observed between variables indicating cognitive and motor inhibition within the BRTs, except for a single low, but non-significant correlation out of six in each age group. Furthermore, neither significant associations nor moderate correlations were observed between the overall performance between stepping and gait-initiation tasks, indicating a disparate structure.

### Cognitive functions partly account for performance of the two balance-related tasks

In addition to processing time (reaction time in the Go/no-go test), basic processing speed and visual perceptual ability, as measured by TMT A, significantly contributed to overall performance in the stepping task, while cognitive flexibility and working demands, assessed by TMT difference^51^, was a significant predictor of overall performance in the gait initiation-stop task. Although the latter BRT ultimately relies on “pure” postural control to successfully maintain balance and remain standing, the gait initiation-stop task may have demanded more cognitive control than the stepping task because of the critical role of higher-order executive functions in perceiving and correcting instability. In addition, cognitive flexibility closely intercorrelates with inhibition, to the extent that it integrates inhibition as part of its broader adaptive control processes^52,53^.

Variation in these predictors, could explain 62% and 56% of the variation in the overall outcome of each BRT in YA (*R²* for the stepping task: 0.62 (bootstrap 95% CI: 0.26, 0.84) and for the gait initiation-stop task: 0.56 (bootstrap 95% CI: 0.04, 0.55)). Although R-squared is a useful measure of model ‘goodness of fit’^32^, these findings should be interpreted with caution, as R-squared is strongly influenced by variance in the population, with higher variance potentially inflating R-squared^54^.

### Findings in older adults

Findings in OA were not consistent with the features of *convergence*,* divergence*, and *disparate structure* in the BRTs as observed among YA. The lack of consistent associations between the general inhibition tests and the BRTs, along with the less predictable performance of the BRTs performance observed in OA, may be related to cognitive compensatory processes and age-related changes in brain activity. Neurophysiological studies of general inhibition tests indicate that, unlike YA, OA do not exhibit distinctly prominent brain activity associated with inhibitory control^55,56^. Instead, inhibitory control-related activity occurs in conjunction with more widespread neural activation^56,57^. The two novel BRTs simultaneously demand cognitive functions (amongst which inhibition), balance control and the preparation (and execution) of step responses, which may elicit an even larger neural engagement than general inhibition tests, potentially leading to a diffusion of resources. Thus, OA may need to allocate executive and physical resources more broadly, reducing their efficiency and consistency. One theory, known as ‘*dedifferentiation***’**, explains age-related changes in the brain by postulating a reduction in activity within task-specific areas in OA^58^. As a result, OA rely on broader activation of brain regions, which may hinder processing speed and reduce neural efficiency. Another theory addressing the same phenomenon from a different perspective is the ‘*scaffolding theory* of aging and cognition’^59^. This theory posits that functional and structure declines in the aging brain are compensated through the recruitment of alternative neural networks, which act as a compensatory mechanism to support cognitive functions. Nevertheless, it is worth noting that none of the variables from the general inhibition tests showed even a meaningful tendency towards relations, including inhibitory initiation time during step initiation (CI CoP onset). This may reflect that reaction time under balance-control demands is affected differently than reaction time measured during seated finger responses, particularly in OA.

Consistent with previous reports of more pronounced age-related declines in motor inhibition than cognitive inhibition^5,60^, our findings reveal that cognitive inhibition during the BRT did not exhibit statistically significant age-related differences, whereas motor inhibition did. This suggests differing degrees of age-related changes in cognitive and motor inhibition. Furthermore, our findings could not identify a stable predictive model for motor inhibition in the BRT in OA. The lack of predictability may possibly be caused by less consistent behavior of OA and an inability to compensate for reduced inhibitory control by other executive or general physical functions, further highlighting the task-specific features of motor inhibition during the BRT.

### Strengths and weaknesses

To the best of our knowledge, this is the first study to investigate different aspects of inhibitory control, as integrated into two BRTs, as well as general tests, assessing executive and physical functions, within the same participants, comprised of young and older groups. Our findings make an important novel contribution to understanding relations between inhibitory control during balance-related and conventional inhibition tasks performed while sitting, while also examining age-related changes. However, given the limited sample sizes, our exploratory analyses considered the magnitude of correlations as indicators of effect size rather than relying solely on statistical significance, aligning with Cohen’s recommendations^29^. Furthermore, OA included in this study are not representative for the general older population, given their relatively higher levels of physical activity and low fall rates. Accordingly, similar studies with a larger sample size, particularly in YA and in specific groups of OA who are less fit and active are needed to confirm our results and demonstrate the reliability of these findings.

### Conclusion and outlook

Our findings in YA give evidence of task specific aspects of inhibitory control in the two BRTs. The absence of similar results in OA may reflect cognitive compensatory processes. These behavioral findings provide a valuable contribution to further foundation for further research exploring the neurophysiological mechanisms underlying age-related changes in inhibitory control integrated into BRTs. Furthermore, our findings highlight the need of novel approaches to understand and assess individual performance in complex balance-related tasks. While widely used to assess fall risk^61^, the Timed-up & go test and gait speed may not fully capture the complex, cognitively demanding situations that can lead to real-world falls. Several studies point to the relevance of balance assessment tools that include cognitive functions for understanding real-life mobility^62,63^ and fall risk^64,65^. Nonetheless, no widely adopted balance task currently incorporates cognitive functions in a way that mimics everyday life. Notably, even healthy older adults in the present study demonstrated age-related differences in the contributions underlying performance on novel balance tasks involving cognitive and motor inhibition. This may indicate task-specific adaptive resource structures recruited for each BRT, particularly for the BRT incorporating motor inhibition. Thus, BRTs that mimic aspects of everyday activities, as implemented in the present study, may be valuable for evaluating individual performance and assessing fall risk, providing a foundation for the development of diagnostic tools tailored to older populations.

## Supplementary Information

Below is the link to the electronic supplementary material.

Supplementary Material 1

Supplementary Material 2

## Data Availability

Data supporting the conclusions of this article will be made available upon request to the corresponding author.

## Code availability

The essential code for the analysis of both of our balance tasks comprised the timing of visual stimuli and the detection of changes in the CoP signal. The primary MATLAB/Psychtoolkit scripts used to generate the results are publicly available at Zenodo (DOI: 10.5281/zenodo.18880098). Further implementation details can be provided upon reasonable request.
