# Supplementary material for: Cognitive and motor inhibition in balance-related tasks: task-specific associations with executive and physical functions in young and older adults
Source: Sci Rep. 2026 Mar 17;16:9234. doi: 10.1038/s41598-026-44189-x (PMC13000256; doi:10.1038/s41598-026-44189-x)
Supplement: Supplementary file 1 — Supplementary Material 1 [file 41598_2026_44189_MOESM1_ESM.pdf]

Title: **Cognitive and motor inhibition in balance-related tasks: task-specific associations with executive and physical functions in young and older adults**

Journal: **Scientific Reports**

Authors: Eunyoung Kwag*[1], Dr., Wiebren Zijlstra[1], Univ.-Prof. Dr.

Affiliation: [1]Institute of Movement and Sport Gerontology, German Sport University Cologne, Cologne, Germany

Corresponding author (*)

Eunyoung Kwag, Institute of Movement and Sport Gerontology, German Sport University Cologne, Am Sportpark Müngersdorf 6, 50933, Cologne, Germany

E-Mail: en.kwag@gmail.com

Appendix1.

Tests assessing executive functions:

(1) Inhibitory control evaluated through web-based tests, including Go/No-go test and Stop-signal test

- **Go/no-go test** [1]: a participant is seated comfortably at a computer, with the index finger of their preferred hand resting on a spacebar. When a green ‘Go’ stimulus appears on the screen, the participant is instructed to press the spacebar as quickly as possible. However, when a red ‘No-go’ stimulus is presented, the participant is required to withhold any response.

A familiarization block incorporating feedback, consisting of 15 ‘Go’ and 5 ‘No-go’ trials, is followed by two main blocks, each containing 64 ‘Go’ and 16 ‘No-go’ trials (20 % ‘No-go’ trials) without feedback. For additional details regarding trial timing, see Figure 1.

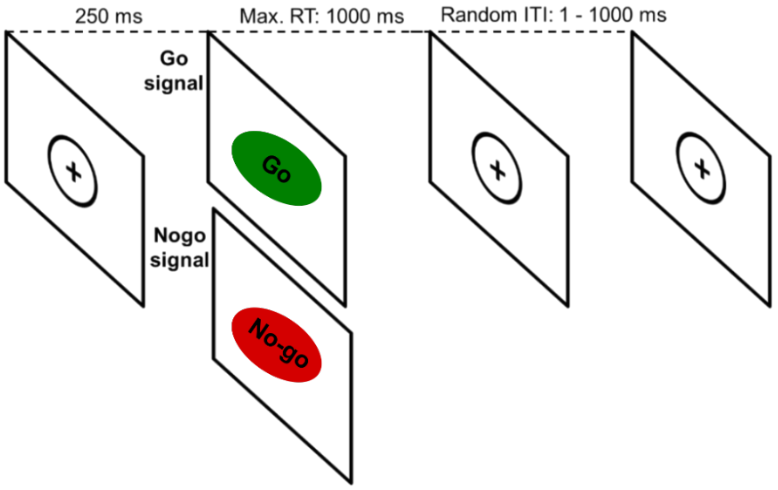

Fig.1 Go/no-go test procedure

Note. *ITI*, inter-trial interval; *Max,* maximum; *RT,* reaction time

- **Stop-signal test** [2,3]: a participant is seated comfortably at a computer, with their left and right index fingers resting on the ‘b’ and ‘n’ keys, respectively. When a left arrow is presented on the screen, the participant is instructed to press the ‘b’ key as quickly as possible. When a right arrow is presented, the participant is instructed to press ‘n’ key as quickly as possible. However, if the circle surrounding the arrow changes from white to red, the participant is required to withhold any response.

A total of 192 trials were conducted, consisting of three blocks, each containing 48 ‘Go’ trials and 16 ‘Stop’ trials (25 % ‘Stop’ trials). ‘Stop’ trials were presented using a tracking procedure, with an initial stop signal delay of 250 ms. If the participant failed to stop their response, the delay was reduced by 50 ms in the next trial; if the participant successfully inhibited their

response, the delay was increased by 50 ms. Feedback (‘correct’, ‘do not press’, ‘too late’, and ‘false key’) was displayed for approximately 200 ms following each response. For details on timing, see Figure 2.

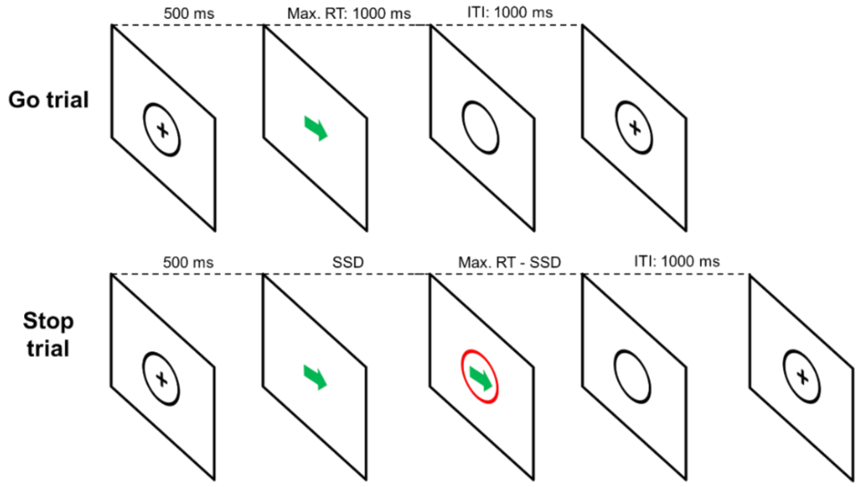

Fig.2 Stop-signal test procedure

Note. *ITI*, inter-trial interval; *Max,* maximum; *RT*, reaction time; *SSD*, stop signal delay

(2) Cognitive flexibility (paper-and-pencil form): Trail making test [4]

The Trail making test consists of two parts, A and B. In part A, a participant is required to connect encircled numbers in ascending order from 1 to 25 as quickly as possible. In part B, the participant is required to connect circles while alternating between numbers and letters in ascending order (e.g., 1, A, 2, B) up to 13, completing the sequence as quickly as possible. If an error is not self-corrected, examiner prompts the participant to make the correction. Total completion time is recorded using a stopwatch, and the number of errors is documented. Both parts were administered following a brief practice session for each.

(3) Working memory (tapping cubes): Corsi block test [5,6]

The Corsi block test was administered using a physical 3D block structure as described by Kessels et al. (2000) (see Figure 3). A participant and an examiner sat opposite each other, with the examiner initiating the test by tapping a sequence of two blocks. The participant then replicated the sequence by tapping the same blocks in the same order. Each sequence length was presented twice, and if correctly reproduced, the sequence length was increased. Both forward and backward recall conditions were assessed. Performance was measured by span (the number of blocks tapped), the number of correct responses, and product score (span x number of correct responses).

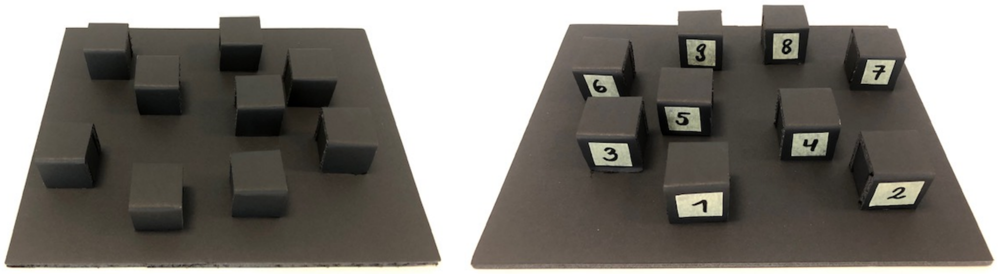

Fig.3 Physical 3D Corsi block structure used in this study (Photo taken by Dr. Eunyoung Kwag) The left image presents participant's perspective, while the right image shows examiner's view Note. *ITI*, inter-trial interval; *Max,* maximum; *RT*, reaction time; *SSD*, stop signal delay

Tests assessing physical functions:

(1) Balance test: The Berg balance scale [8]

The Berg balance scale is a validated and reliable tool used to assess functional balance. It includes 14 tasks that evaluate various aspects of balance, such as static sitting, standing, turning, and other movements essential for maintaining stability.

(2) Mobility test: The modified Timed-up & go at normal and fast speed (walk a distance about 6 meter)

In **the Timed-up & go test**, a participant is asked to rise from a chair with their arms crossed in front of their chest, walk to a marker placed 6 meters away, turn around, walk back, and sit down again in the starting position. The participant is required to walk at normal speed, followed by at fast speed (but not running). Each condition is repeated three times.

(3) Balance confidence test: The Activity-specific balance confidence scale [9]

The Activity-specific balance confidence scale is a self-report questionnaire that measures confidence in performing balance-related activities, comprising 16 items.

References

1. Wessel JR. Prepotent motor activity and inhibitory control demands in different variants of the go/no-go paradigm. Psychophysiology. 2018;55(3):e12871.
2. Verbruggen F, Aron AR, Band GPH, Beste C, Bissett PG, Brockett AT, et al. A consensus guide to capturing the ability to inhibit actions and impulsive behaviors in the stop-signal task. Elife. 2019;8. doi:10.7554/eLife.46323.
3. Band GPH, van der Molen MW, Logan GD. Horse-race model simulations of the stop-signal procedure. Acta Psychol (Amst). 2003;112(2):105–142.
4. Tombaugh T. Trail Making Test A and B: Normative data stratified by age and education. Arch Clin Neuropsychol. 2004;19(2):203–214.
5. Arce T, McMullen K. The Corsi Block-Tapping Test: Evaluating methodological practices with an eye towards modern digital frameworks. Comput Hum Behav Reports. 2021;4:100099.
6. Kessels RPC, Van Den Berg E, Ruis C, Brands AMA. The backward span of the corsi block-tapping task and its association with the WAIS-III digit span. Assessment. 2008;15(4):426–434.
7. Kessels RPC, Van Zandvoort MJE, Postma A, Kappelle LJ, De Haan EHF. The Corsi Block-Tapping Task: Standardization and normative data. Appl Neuropsychol. 2000;7(4):252–258.
8. Berg K, Wood-Dauphinee S, Williams J. The Balance Scale: reliability assessment with elderly residents and patients with an acute stroke. J Rehabil Med. 1995;27(1):27–36.
9. Powell LE, Myers AM. The Activities-Specific Balance Confidence (ABC) scale. Journals Gerontol - Ser A Biol Sci Med Sci. 1995;50A(1):M28–M34.
