# Supplementary material for: Cognitive and motor inhibition in balance-related tasks: task-specific associations with executive and physical functions in young and older adults
Source: Sci Rep. 2026 Mar 17;16:9234. doi: 10.1038/s41598-026-44189-x (PMC13000256; doi:10.1038/s41598-026-44189-x)
Supplement: Supplementary file 2 — Supplementary Material 2 [file 41598_2026_44189_MOESM2_ESM.pdf]

Title: **Cognitive and motor inhibition in balance-related tasks: task-specific associations with executive and physical functions in young and older adults**

Journal: **Scientific Reports**

Authors: Eunyoung Kwag*[1], Dr., Wiebren Zijlstra[1], Univ.-Prof. Dr.

Affiliation: [1]Institute of Movement and Sport Gerontology, German Sport University Cologne, Cologne, Germany

Corresponding author (*)

Eunyoung Kwag, Institute of Movement and Sport Gerontology, German Sport University Cologne, Am Sportpark Müngersdorf 6, 50933, Cologne, Germany

E-Mail: en.kwag@gmail.com

Appendix 2.

Comprehensive correlations between the balance-related tasks and general tests for young and older adults

	**YA**	**Step**	**Step**	**Gait**	**Step**	**Gait**
		**CI CoP onset**	**CI CoP duration**	**MI StopGo integrals**	**Total step execution (ms)**	**Success (%)**
Balance-related tasks	**CI CoP onset**	1.000	-0.183	-0.143	***0.498***	0.244
	**CI CoP duration**	-0.183	1.000	**-0.407**	**-0.309**	0.060
	**MI StopGo integrals**	-0.143	**-0.407**	1.000	0.262	**-0.417**
	**Total step execution (ms)**	***0.498***	**-0.309**	0.262	1.000	0.032
	**Success (%)**	0.244	0.060	**-0.417**	0.032	1.000
Inhibition tests	**Reaction time (ms)**	***0.719***	**-0.449**	0.042	***0.503***	**0.463**
	**Failure (%) in GNG**	-0.246	***0.639***	-0.207	**-0.380**	-0.142
	**SSRT (ms)**	-0.162	-0.117	**0.400**	**0.302**	0.038
	**Failure (%) in SST**	**-0.409**	***0.478***	0.185	-0.125	0.187
Other executive functions test	**TMT Difference (s)**	0.166	-0.172	0.231	**0.391**	***-0.753***
	**TMT A (s)**	-0.017	0.073	-0.204	**-0.340**	0.025
	**TMT B(s)**	0.073	-0.077	0.046	0.059	***-0.637***
	**Forward CBT**	0.028	**-0.385**	-0.023	0.079	0.180
	**Backward CBT**	-0.119	**-0.334**	0.113	-0.188	-0.100
Physical functions test	**Normal TUG (s)**	**0.313**	0.150	-0.182	**0.346**	0.028
	**Fast TUG (s)**	0.164	-0.120	0.138	***0.494***	-0.226
	**ABC (%)**	**0.358**	0.074	0.013	0.143	0.036

	**OA**	**Step**	**Step**	**Gait**	**Step**	**Gait**
		**CI CoP onset**	**CI CoP duration**	**MI StopGo integrals**	**Total step execution (ms)**	**Success (%)**
Balance-related tasks	**CI CoP onset**	1.000	-0.134	-0.281	-0.120	-0.020
	**CI CoP duration**	-0.134	1.000	0.036	-0.098	-0.057
	**MI StopGo integrals**	-0.281	0.036	1.000	**0.330**	***-0.382***
	**Total step execution (ms)**	-0.120	-0.098	**0.330**	1.000	-0.064
	**Success (%)**	-0.020	-0.057	***-0.382***	-0.064	1.000
Inhibition tests	**Reaction time (ms)**	-0.278	0.007	0.186	***0.415***	-0.126
	**Failure (%) in GNG**	-0.267	-0.192	0.090	0.096	0.014
	**SSRT (ms)**	-0.127	-0.126	0.170	***0.335***	0.015
	**Failure (%) in SST**	0.142	-0.116	0.139	0.186	-0.206
Other executive functions test	**TMT Difference (s)**	-0.074	0.188	0.179	-0.060	-0.131
	**TMT A (s)**	0.130	***-0.329***	0.083	0.200	-0.231
	**TMT B(s)**	-0.057	0.083	0.208	-0.040	-0.147
	**Forward CBT**	0.262	**0.308**	-0.111	-0.096	-0.067
	**Backward CBT**	0.039	0.166	0.258	0.145	-0.124
Physical functions test	**Normal TUG (s)**	-0.142	***-0.403***	**0.334**	0.104	0.021
	**Fast TUG (s)**	***-0.393***	-0.237	***0.355***	0.212	-0.023
	**BBT**	***0.393***	0.059	***-0.505***	***-0.323***	-0.102
	**ABC (%)**	0.119	-0.043	-0.292	-0.139	0.195

ABC, the Activity-specific balance confidence scale; *BBT*, the Berg balance scale; *CBT,* the Corsi block test; *CI,* cognitive inhibition; *CoP,* Center of pressure; *GNG,* the Go/no-go test; *MI,* motor inhibition; *OA,* older adults; *SSRT,* stop signal reaction time; *SST,* the Stop-signal test; *TMT,* the Trail making test; *TUG,* the Timed-up & go; *YA,* young adults
